# Meta-analytic evidence of low convergence between implicit and explicit measures of the needs for achievement, affiliation, and power

**DOI:** 10.3389/fpsyg.2014.00826

**Published:** 2014-08-08

**Authors:** Martin G. Köllner, Oliver C. Schultheiss

**Affiliations:** Human Motivation and Affective Neuroscience Lab, Department of Psychology and Sport Sciences, Institute of Psychology, Friedrich-Alexander University Erlangen-Nürnberg (FAU)Erlangen, Germany

**Keywords:** implicit motives, implicit-explicit correlation, independence postulate, meta-analysis, motive congruence, picture-story exercise, questionnaire, self-attributed motivation

## Abstract

The correlation between implicit and explicit motive measures and potential moderators of this relationship were examined meta-analytically, using Hunter and Schmidt's (2004) approach. Studies from a comprehensive search in PsycINFO, data sets of our research group, a literature list compiled by an expert, and the results of a request for gray literature were examined for relevance and coded. Analyses were based on 49 papers, 56 independent samples, 6151 subjects, and 167 correlations. The correlations (ρ) between implicit and explicit measures were 0.130 (CI: 0.077–0.183) for the overall relationship, 0.116 (CI: 0.050–0.182) for affiliation, 0.139 (CI: 0.080–0.198) for achievement, and 0.038 (CI: −0.055–0.131) for power. Participant age did not moderate the size of these relationships. However, a greater proportion of males in the samples and an earlier publication year were associated with larger effect sizes.

## Introduction

From the start, research on implicit motives—nonconsciously operating dispositions for seeking certain classes of incentives and avoiding certain classes of disincentives—was built on the notion that people do not have valid introspective access to the motivational forces driving their behavior and that motivation must therefore be measured by means other than self-report (Murray, 1938; McClelland et al., 1953; McClelland, 1984). This has led to the development and validation of thematic apperceptive methods such as the Picture Story Exercise (PSE), which require research participants to write imaginative stories about images depicting a variety of social situations (Smith, 1992). Stories are later coded for motivational imagery according to scoring systems developed through experimental motive-arousal studies (Winter, 1999). From the very beginning, research has documented that motive scores derived in such a manner fail to correlate strongly with the motivational needs that people ascribe to themselves on questionnaire measures (so-called *explicit motive* measures; McClelland et al., 1989a); they also predict different outcomes (e.g., DeCharms et al., 1955). However, because a few studies reported substantial positive correlations between picture-story and self-report measures of motives (e.g., Sherwood, 1966), some researchers have argued against the idea that motives are generally hidden to introspection (e.g., Thrash et al., 2010), and some continue to call for the substitution of labor-intensive picture-story/content-coding methods with more economical self-report measures (e.g., Sokolowski et al., 2000). Given the heterogeneity of empirical findings and the concomitant diversity of conceptual perspectives on the issue of people's introspective access to their motivational needs, we suggest that it is high time to take stock empirically and use the many available studies simultaneously employing picture-story and self-report motive measures to address this issue meta-analytically.

In the present research, we follow McClelland et al.'s (1989a; Weinberger and McClelland, 1990) lead, who argued that behavior is regulated by two independently operating and neurobiologically distinct systems—an implicit, affect-driven, motivational system and an explicit, self-concept-related system representing a person's values, beliefs, and goals (for similar arguments, see also Rolls, 1999; Berridge and Robinson, 2003; Brunstein, 2008). To the extent that McClelland et al.'s (1989a) arguments are valid, it follows that measures of the implicit system should not converge, or have substantial overlap, with measures of the explicit system, because they represent fundamentally different modes of shaping behavioral output. This has been called the *independence postulate* (Schultheiss et al., 2009) and suggests that, overall, correlations between implicit and explicit motivation measures should be correlated close to zero, as McClelland et al. (1989a) argued.

But how close to zero lies true independence and where does substantial variance overlap between two measures start? First of all, we do not propose that measurements of any two factually independent attributes, across many studies, are likely to ever yield a meta-analytic association of exactly zero. Thus, the issue of independence becomes one of degree—how small is small enough to support the notion of independently operating systems? For standards of meta-analytic comparison, we turn to two meta-analyses which, like ours, compare self-report measures with procedural measures of a target attribute. Cyders and Coskunpinar (2011) ran a meta-analysis of 27 published studies of the association between self-report and behavioral laboratory task measures of impulsivity. They found the average correlation between the two types of measures was ρ = 0.097, corresponding to shared variance of less than 1%. Cyders and Coskunpinar (2011) classified this as very little overlap (p. 980). They concluded that these findings “[…] suggest that these disparate measures should not be referred to under the same rubric of impulsivity […]” (p. 980), echoing McClelland et al.'s (1989a) similar conclusion for the domain of motivation research that “[…] psychologists should not call by the same name two measures that do not correlate with one another […]” (p. 691). Hofmann et al. (2005) conducted a meta-analysis of 126 studies using self-report and implicit-association test (IAT; Greenwald et al., 1998) measures of attitudes. Across studies, they found an average correlation of 0.24 between the two types of measures, which they classified as “small” (p. 1379).

While we would view an effect size of 0.24 as perhaps too high for holding fast to a notion of independence, we agree with the conclusion by Cyders and Coskunpinar (2011) that an effect size of around 0.10 indicates de-facto independence between two types of measures. Spangler (1992) published the only meta-analysis on implicit and explicit motives so far, focusing exclusively on the domain of achievement motivation. He examined the convergence between picture-story and self-report measures in 36 correlations and found a small, but statistically significant average correlation of 0.088. This is much closer to the benchmark by Cyders and Coskunpinar (2011) than to the one by Hofmann et al. (2005) and represents the effect size that we expected to find in our present research.

Given the heterogeneity of published correlations between implicit and explicit motive measures, however, it appears likely that even if we can replicate and extend Spangler's (1992) meta-analytic findings to motivational domains other than achievement, moderators may influence effect sizes. One moderator may be the content domain of motivation itself. The three domains we focus on in this study are the need for achievement (often abbreviated *n* Achievement), a capacity for gaining pleasure from mastering challenging tasks; the need for affiliation (or *n* Affiliation), a capacity for deriving pleasure from close, friendly relationships with others; and the need for power (*n* Power), a capacity for getting pleasure out of having impact on others (see Schultheiss, 2008; for alternative conceptual frameworks of human motivation, see Bakan, 1966; Deci and Ryan, 2000). Among other possible factors, social desirability may influence the correlation between self-report and picture-story measures of motives to varying degrees. While *n* Achievement and *n* Affiliation clearly are desirable attributes in our society, *n* Power, carrying connotations of dominance and even aggression, may be not (cf. McClelland, 1987). If one adopts the view that there may be parts of implicit motives accessible to introspection (see Fineman, 1977), then social desirability may lead to concealing *n* Power, but not other motives, in questionnaires, reducing the observed correlation (cf. McClelland, 1987; Thrash et al., 2010). We will explore the validity of this assumption by comparing the correlations of socially desirable motives like *n* Achievement and *n* Affiliation with those for the less desirable *n* Power.

We also explored methodological aspects (cf. Hofmann et al., 2005) as moderators, such as characteristics of the implicit and the explicit measure, their order in the study design, sample age and gender composition, and publication format and year of the studies.

To replicate and extend the results of Spangler's (1992) meta-analysis and investigate the influence of some moderator candidates, we conducted our meta-analysis using Hunter and Schmidt's (2004) approach. Studies were retrieved from several sources, filtered, and coded. We chose a bias-free, explorative approach and objective criteria. As stated by Rosenthal and DiMatteo (2001), quantitative reviews are better at detecting relationships if they choose an explorative instead of a confirmatory perspective. Thus, the hypotheses below were formulated to facilitate data interpretation only and not to restrict our exploratory approach.

Generalizing Spangler's (1992) results for *n* Achievement across all three motive domains, we expected a small positive overall correlation of implicit and explicit motive measures. Based on Spangler's (1992) study, we expected the confidence interval (CI) of the estimated mean population correlation ρ to exclude zero (*Main hypothesis 1*).

For *n* Achievement, we expected to replicate the small positive correlation of implicit and explicit motive measures reported by Spangler (1992), with the CI around ρ including his estimate of *r* = 0.088 and excluding zero (*Main hypothesis 2*).

Because *n* Affiliation, like *n* Achievement, is a socially desirable disposition and there should be no incentive to conceal it, we expected a small positive correlation of implicit and explicit measures for *n* Affiliation, too. Thus, the CI around ρ should not include zero (*Main hypothesis 3*).

Following McClelland's (1987) lead, we regarded *n* Power as a socially undesirable disposition. Thus, what little insight people may have into their motive dispositions as reflected in their responses to self-report measures should be counteracted in this case by a strong inclination to deny one's *n* Power, a tendency that should not be present or that may even point in the opposite direction for the other two motives. We therefore expected no correlation of implicit and explicit measures of *n* Power. A CI placed around ρ should include zero (*Main hypothesis 4*).

Additionally, we also explored if variability in the population correlation of implicit and explicit motive measures is due to external moderator variables (see Table 1 for an overview). We tested the following potential moderators of the overall relationship, either because we had a plausible assumption for them or because they were basic sample features, like participant age and gender.

**Table 1:** **Overview of moderator candidates of the overall relationship of implicit and explicit motive measures investigated in additional hypotheses**.

**Number**	**Variable**	**Type**	**Additional hypothesis**
1.1	Implicit format	Nominal	Paper and pencil = PC = Oral
2.1	Explicit construct	Nominal	Goals/Wishes > Explicit Motives
3.1	gender	Interval	Female = Male
3.2	mean age	Interval	No correlation
4.1	Order of administration	Nominal	Implicit measure first > Explicit measure first
5.1	Information source	Nominal	Book(chapter)/Journal > Dissertation/Unpublished
5.2	Publication year	Interval	Positive correlation

Considering characteristics of the implicit measure, one methodological issue concerns the data collection format of the PSE (Schultheiss and Pang, 2007). Building on Blankenship and Zoota (1998), who found no substantial differences in *n* Power scores for PC vs. paper-and-pencil administration, we expected the relationship to be independent of the data collection format and thus no reliable differences between PC administration, handwritten PSEs, or other formats (*Additional hypothesis 1.1*).

Among the characteristics of the explicit measure, perhaps the construct on which the explicit measure is based makes a difference. Some have argued that measures of specific personal goals may provide a more direct readout of underlying motivational structures than measures of generalized explicit values or self-attributed motives, because the former are thought to be more strongly influenced by motives than the latter (e.g., Emmons, 1989; but see Rawolle et al., 2013). We therefore expected the relationship between implicit and explicit measures to be stronger if, at the explicit level, specific wishes or goals are examined as opposed to more general motivational orientations *(Additional hypothesis 2.1.)*.

We examined the roles of gender and age, two basic sample characteristics. As we did not know of any study directly testing gender differences of the relationship, we expected no difference between women and men (*Additional hypothesis 3.1*.). Furthermore, we adopted McClelland et al.'s (1989a) position that the convergence between implicit and explicit motives does not increase across the life span. We therefore expected the relationship to be independent of the mean age of the samples (*Additional hypothesis 3.2*.).

Study design or study procedure may exert an influence on the results, too. Hofmann et al. (2005) investigated order of measures as a potential moderator. In our case, Schultheiss and Pang (2007) stated that other preceding measures may distort the PSE's validity, and we therefore assume that putting the explicit measure before the implicit picture-story measure may lead to hypothesis guessing and distort motive expression in the PSE. We therefore expected the relationship to be stronger if the implicit preceded the explicit measure than in the reverse case (*Additional hypothesis 4.1*).

A final issue is the origin of studies. Hunter and Schmidt (2004) consider unpublished material to produce lower effect sizes than published studies. Building on a classification based on four different study sources (Rosenthal, 1991), we expected a stronger relationship for book chapters or journal articles than for dissertations (or other theses) or unpublished material, but no difference between chapters and articles or between theses and unpublished material, respectively (*Additional hypothesis 5.1*.). Furthermore, as motive measurement might have improved over time through repeated revisions of coding systems and more sophisticated approaches to test construction, possible conscious aspects of the implicit motive (see Fineman, 1977) might have a better chance of being captured by both the implicit and the explicit measure, enhancing the correlation. The relationship will be positively correlated with year of publication of a study (*Additional hypothesis 5.2*.).

## Materials and methods

**Figure 1:**
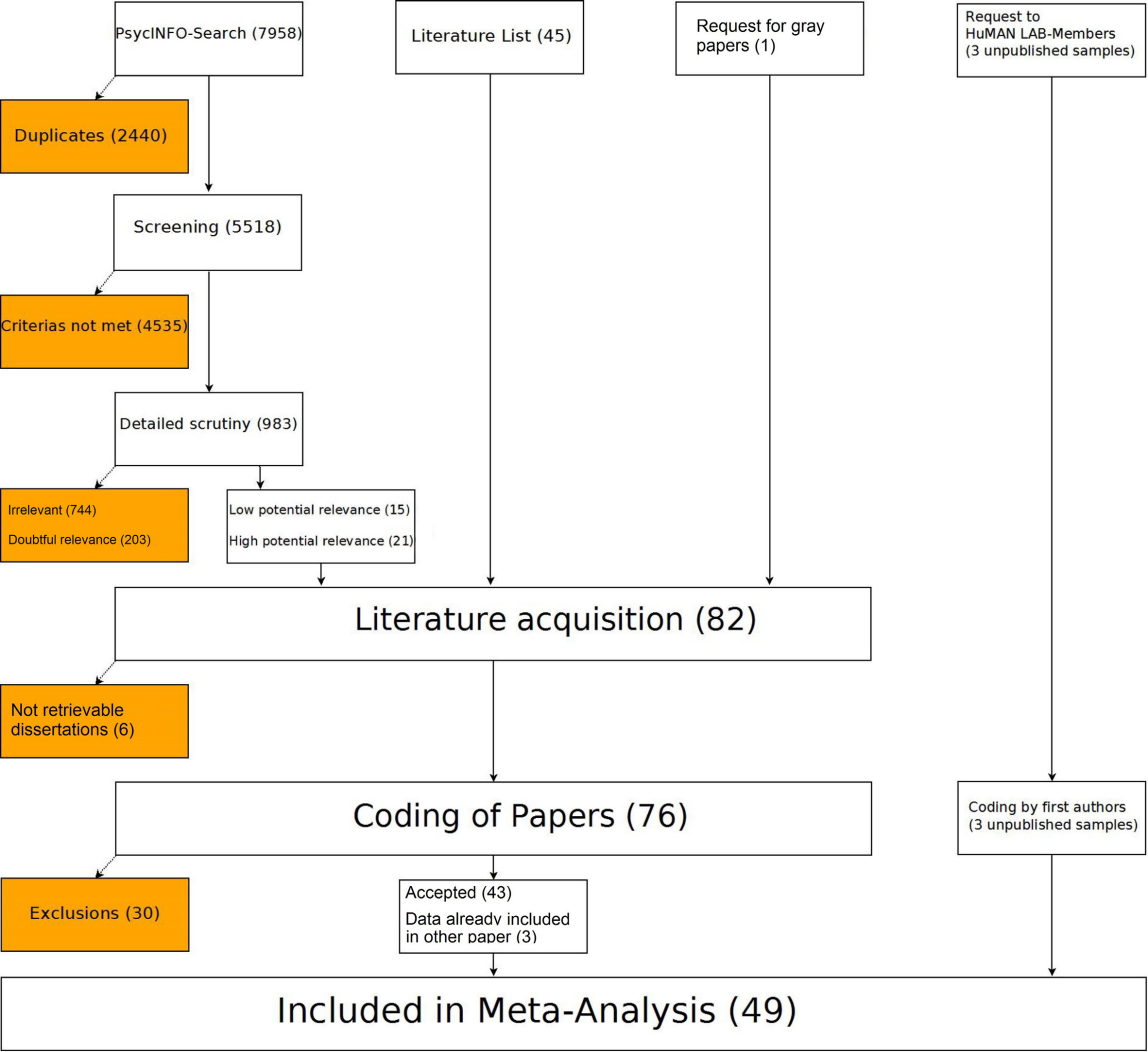
**Overview of the procedure used in collecting and analyzing the literature with number of studies in the respective subsample given in brackets**. White background, retained; dark background, excluded.

Literature was gathered and analyzed in three steps prior to meta-analytic computations: A comprehensive literature search, followed by acquisition and coding of eligible papers (for an overview see Figure 1). Units of analysis were studies meeting the inclusion criteria specified below. We use the term “study” to refer to a statistically independent sample, not to a paper.

### Inclusion criteria

The literature search included journal articles, books or book chapters, dissertations and other theses, as well as unpublished data. We limited the time span of our search to the interval between 1953—the year in which McClelland et al. (1953) published the field-defining volume on *n* Achievement—and April 2009, marking the end of our literature search. There were no restrictions concerning populations, geographical location, culture, study design, or sample size.

However, studies had to fulfill the following criteria to be eligible for the meta-analysis:

1. As an implicit measure, a study had to include a picture-story instrument combined with the application of empirically derived (see Winter, 1999) coding systems for at least one of the following motives: achievement, affiliation, or power. We regarded coding systems as empirical mainly if they were reported in Smith (1992). Winter's (1994) running-text system and Heckhausen's (1963) system for the assessment of hope for success and fear of failure were also included. Studies using verbal cues instead of pictures cues (Karabenick, 1977; Gelbort and Winer, 1985; Langan-Fox, 1991; Hofer et al., 2006a) were eligible for inclusion, too, because they used the same empirically derived content coding systems for scoring stories.
2. In addition, an explicit motive measure including self-report or self-description in any form was required. Eligible measures ranged from rating scales to idiographic self-reports. Measures needed not to be described as assessing “self-reported motives,” as long as the explicit measures roughly corresponded to the content of the implicit motive measures. Motivation, values, preferences and similar labels were eligible, if the items or the scoring of the texts met this criterion. In sum, eligible measures included the whole spectrum of measures from questionnaires like Personality Research Form (PRF; e.g., Jackson, 1999) or Edwards Personal Preference Schedule (EPPS; e.g., Edwards, 1954) to measures assessing personal goals, wishes, etc. Considering valence, we included only correlations between similarly oriented measures to ensure correspondence to the content of the implicit coding system. For instance, a correlation between implicit and explicit fear of failure would have been eligible, but not one between explicit fear of failure and implicit hope for success.
3. Implicit and explicit measures had to be reported quantitatively to prevent the loss of information and power accompanying dichotomization (Cohen, 1983). In addition to Pearson correlations, alternative correlation coefficients like Spearman were also eligible.
4. Correlations had to be reported precisely. Studies without exact values were excluded. Three studies reported correlations selectively, focusing perhaps only on unusually high or low effect sizes: One only reported significant correlations for single pictures and therefore was excluded (Evans and Singer, 1994). Two studies investigated several motives, but reported exact data on one motive only because of a significant or almost significant correlation, while not reporting exact data on the remaining motives for which data also had been collected (McClelland et al., 1998; Hofer and Chasiotis, 2003). To retain comparability of main and moderator analyses and to ensure that all analyzed studies stemmed from the same pool as the ones in the overall analyses, these two studies were not included in the main and moderator analyses. However, acknowledging the valuable contributions to the field made by those studies, they were flagged and retained, but only used in two additional analyses mentioned under “Results for untransformed, uncorrected correlations.”
5. To prevent duplication, data already coded out of one paper were not coded twice if they reappeared in other publications.
6. We used only papers in English or German language to avoid mistakes through imprecise translation.

### Literature search

Literature was collected with four different methods: A literature list compiled by the second author (“positive list,” see below), a comprehensive online-search in PsycINFO, a request to members of the “Human Motivation and Affective Neuroscience Lab” (HuMAN Lab) at Friedrich-Alexander University, and a request for unpublished literature to the mailing list of the Society for Personality and Social Psychology (SPSP).

#### Positive list

Based on his research expertise in motivational psychology, the second author compiled a list of 45 papers for which relevance to our topic was assumed. These papers were all included a priori in the coding process. Another purpose of this list was to gauge the validity of our literature searches—search results were deemed valid to the extent that they identified, among others, the papers contained in the positive list.

#### Search in PsycINFO

We searched PsycINFO for terms or term combinations generated from filtering keywords out of papers from the positive list and from key articles (e.g., McClelland, 1980; McClelland et al., 1989a; King, 1995) and further supplemented and modified them. We then generated keyword combinations like the term collections below intended to illustrate the type of words used:

“(thematic apperception or thematic analysis or picture story or TAT or picture-story or picture-story exercise or Picture Story Exercise or PSE) and (explicit measur^*^ or questionnaire or personality scal^*^ or inventory or self-ratin^*^ or self-repor^*^ or self-evaluation or personal strivin^*^ or goal or Personality Research Form or PRF or California Psychological Inventory or CPI or EPPS or Edwards Personal Preference Schedule),” or

“implicit and (explicit or self-attributed or self-rated or self-reported) and (motiv^*^ or achievement or affiliation or affiliation-intimacy or dominance or intimacy or nurturance or power).”

For every search, we recorded term combination, date, and database coverage^1^ of possible studies. Our search also covered dissertations published by “ProQuest Dissertations & Theses.” Altogether, we searched for about 430 terms or term combinations (for an exhaustive list of these terms, please see the supplementary materials). We exported the hits for every search to Endnote 11.

The results of the PsycINFO search contained all papers from the positive list except for three which were not listed in PsycINFO at all.

#### Screening of search results

The online-search yielded 5518 references, too many for detailed scrutiny. Therefore, we excluded clearly irrelevant items through several screening steps in Endnote. First, we excluded 272 studies conducted before 1953. Then, we ran three combined search steps. Each time, key words were searched in *Any Field* (excluding the *Notes*-field which contained irrelevant information) and combined by *or*. The first step aimed at eligible implicit measures using the words *thematic apperception test, TAT, picture story exercise, picture-story exercise, PSE, thematic apperception* and *content coding*. The next step used the terms *questionnaire, self-report, self-report, scale, rating, survey, inventory, explicit* and *conscious* to identify explicit measures in the remaining entries. Finally, we searched for the motive-domain names *achievement, affiliation, affiliation-intimacy, intimacy, intimacy-affiliation*, and *power* and removed entries already included in the positive list. After this filtering step, 983 references remained.

***Detailed scrutiny of title, abstract and keywords***. The remaining references were examined by the first author. He checked the fields *Title, Keywords*, and *Abstract* for hints at implicit and explicit measures and for data on their relationship and then marked each reference as *irrelevant, doubtful relevance, low potential relevance* or *high potential relevance*. Each reference was checked twice. This was necessary because the inconsistent terminology in the references made it impossible to specify an exhaustive list of words that would have led to inclusion automatically. References were marked *irrelevant* if they did not include motive measures at all or either implicit or explicit measures only, if their focus was not empirical, if they were not written in English or German, or if relevant fields, such as *Abstract*, were empty and there was no clue for relevance in the remaining fields. A total of 744 papers marked as *irrelevant* was deleted.

The label “*Doubtful relevance*” was given to references that possibly fit our topic, but for which clear doubts remained. These doubts could be due to the references containing only minimal clues for eligibility, for example reporting the use of “implicit and explicit measures” without referring to motives or motivation, or the investigation of “implicit and explicit cognition.” Also, references using questionnaires that did not fit our definition of explicit motives belonged to this category. The respective 203 papers were deleted.

*Low potential relevance* included references containing clues for implicit and explicit motive measures. Criteria for detecting implicit measures were use of the acronyms TAT or PSE, the unabbreviated instrument names, or terms indicative of this method like “content coding” or “implicit motives.” Criteria for detecting explicit measures were terms like “self-report measure(s),” “explicit/self-attributed motive(s),” “rating scale,” “personality inventory,” “questionnaire,” or the mention of well-known measures, such as the PRF or the EPPS. Papers with more abstract hints like “conscious and unconscious motives” were included here, too. Fifteen references falling under this rubric were retained.

*High potential relevance* denoted papers obviously dealing with the relationship between implicit and explicit motive measures. It also included references meeting the criteria for *low potential relevance* which in addition included hints at a relationship between implicit and explicit motives through terms like “(un)correlated,” “compared,” “congruence,” or “relation(ship) of.” All 21 respective references were retained.

From the PsycINFO search, only the 36 papers marked as *low* or *high potential relevance* were retrieved (note that these were all papers in addition to those already contained in our positive list). We considered them, along with references found through our three other sources, sufficient to provide a robust approximation of the overall correlation between implicit and explicit motive measures.

#### Request for gray papers

A general validity threat to meta-analyses is a *publication bias* favoring significant findings (Hofmann et al., 2005). Therefore, we submitted a request for gray papers (unpublished research or technical reports, posters or new articles accepted for publication) to the mailing list of the SPSP (spsp@cornell.edu) on March 7 2008. This yielded one dissertation (Fleeson, 1992).

#### Request to HuMAN lab members

An emailed request to the members of the HuMAN Lab on October 26 2009 yielded three independent samples from projects unpublished at that time. The principal investigators associated with each project entered their data in the coding form described below. Data was checked after submission and ambiguities removed with the help of the principal investigators.

#### Literature acquisition

From 82 papers retained after the literature search, we acquired all journal articles, books, or book chapters using PsycINFO, Google, and various online databases or library services. Some dissertations (*n* = 15) could not be retrieved this way: Six were either published online or found in German libraries, provided by the author, or we already owned a copy of them. For the other US American dissertations, we tried to contact the authors, advisors, or institutes. For every reference, we tried to make contact at least once, thereby acquiring three additional references. In the end, nine of 15 dissertations were retrieved. In two cases, the authors mentioned a published version, and so we referred to it instead of the dissertation.

### Coding procedures

The first author coded the 76 remaining papers, excluding papers not meeting our criteria and entering the data of eligible ones into a coding form.

#### Coding form development

The coding form^2^ was based on general recommendations for conducting meta-analyses (Rosenthal, 1991; DeCoster, 2004; Roberts et al., 2007). An identification code was assigned to every correlation derived from a paper's identification number in our database, the independent sample in the paper, and the specific correlation in this sample.

The coding form contained 61 variables organized in different sections: origin (e.g., authors and year of publication), sample information (e.g., sample size, gender composition, and mean age), features of the motive measures (e.g., coding system or number of pictures for the implicit measure; questionnaire and scale name or the construct assessed for the explicit measure), study characteristics (e.g., order of administration of the measures or possible experimental manipulations before or during measurement), and effect size information (e.g., numerical value of the correlation and correlation type, like Spearman or product-moment).

#### Reliability assessment

The first author's coding of a subsample of 15 studies^3^ (20% of the 76 coded papers) was compared to that of an independent second coder. Only papers containing codeable data were used (cf. Roberts et al., 2007). The subsample contained seven papers selected because of their diversity and high coding difficulty. The other eight texts were drawn from the remaining papers and intended to reflect basic database characteristics: One paper from each decade from the 50s to the 80s was included and two from the last two decades each because of their larger quantity. Rare sources were included among them because of possible special reporting features: one book chapter (DeCharms et al., 1955) and a published sub-study of a dissertation (Craig et al., 1994) whose text was almost identical to that in the thesis (Craig, 1996).

The second coder was a student of psychology working at the HuMAN Lab. She was knowledgeable in motivation research in general and in implicit motive assessment specifically. Coding form and manual were explained to her in a training session. Besides independently coding the papers, we recorded reasons for coding decisions in cases of uncertainty and discussed complex cases, thereby creating additional coding rules.

Overall, inter-coder reliabilities were good to perfect. The 15 publications included 53 single correlations (31.74% of all cases). Of the 61 variables, three for which no data were given in the subsample were excluded from reliability assessment. Excluded variables were not used in any analysis. For 16 quantitative variables, we obtained an average Pearson-correlation of *r* = 1.00. For 42 categorical variables, we computed an average percentage agreement of 98.16%, (*SD* = 3.85), with agreements on single variables ranging from 84.91 to 100%, corroborating Hunter and Schmidt's (2004) argument that for objective study characteristics coding reliability is typically very good to almost perfect.

#### Coding procedures

Variables analyzed in the results section^4^ were coded as follows (inter-coder reliability in brackets):

***Effect size (correlation)***. Correlation coefficients (*r* = 1.00) were transcribed for each motive domain from the papers with a precision of three decimal places. This effect size measure was reported in every included study and never had to be computed from other parameters.

***Sample size (N)***. *N* (*r* = 1.00) was the number of subjects on which a correlation was based. Sometimes *N* was not identical to total subject number or varied among different correlations in a study. In rare cases, *N* was computed from other data (e.g., by adding sub-group *n*s to get *N*).

***Motive domain***. Motive domain (100%) specified whether a correlation coefficient was reported for *n* Achievement, *n* Affiliation, or *n* Power. This information was always given in the text. Affiliation-intimacy and intimacy were classified as *n* Affiliation.

***Reliability of the implicit measure***. Reliabilities of implicit and explicit measures were our indicators of study quality. If possible, reliabilities were extracted directly from the text. For the TAT/PSE, we coded two reliability types: Inter-rater reliability and agreement on practice materials. Inter-rater reliability was assessed with two variables, *inter-rater reliability* (*r* = 1.00) for entering the numerical value and *measure of inter-rater reliability* (100%) for indicating the statistical coefficient, using the categories “Kappa,” “Pearson,” “Spearman,” “Percentage category agreement,” “Not reported clearly” and “Others” for entering the name of unusual coefficients. Agreement on practice materials was coded in a similar fashion, *value of agreement* (*r* = 1.00) was the numerical value and *agreement measure used* (100%) indicated the corresponding statistical coefficient, using the same categories as for *measure of inter-rater reliability*, with the additional category “rank order correlation.” Missing information was usually coded as “not reported clearly.” Only in one case we were able to infer the coefficient. Computations of reliability values were only made from unambiguous information; otherwise, values were coded as “not reported clearly.”

***Reliability of the explicit measure***. We entered the numerical value as *reliability value* (*r* = 1.00) and the coefficient used as *reliability measure* (98.11%), using the categories “Cronbach's Alpha,” “Spearman-Brown,” “Split-Half,” “Test-retest,” “Kuder-Richardson,” “Odd-Even,” “Not reported clearly/No information given” and “Others” for entering unusual coefficient names. Procedures for rare cases in which computations of *reliability value* were necessary were the same as for the implicit measure.

***Data collection format of the implicit measure (implicit format)***. *Implicit format* (100%) had four categories: “paper and pencil,” “PC,” “oral” or “not reported.” In the frequent cases where there was no explicit information in the text, we coded studies before the year 2000 as “paper and pencil,” studies since 2000 as “not reported.” We judged PC-administration before 2000 to be very unlikely and oral administration as so uncommon that authors would mention this explicitly.

***Construct on which the explicit measure is based (explicit construct)***. *Explicit construct* (92.45%) had five categories: “self-reported motive” (including terms like “motivation” or “values”), “wishes,” “goals,” “not reported clearly/not reported” (never actually used in coding) and “others” with an empty line for exact construct specification.

***Mean age (age)***. *Age* (*r* = 0.996) was the mean age of the sample. If possible, we transcribed age directly from the text. In other cases, age was estimated from other statistics. If, for instance, range from 16 to 19 years was given, the midpoint, 17.5, was chosen. If computation was impossible, a missing value was coded.

***Gender***. *Gender* was coded in two variables as number of female (*r* = 1.00) and male (*r* = 1.00) participants in a sample and then transformed into the quantitative proportion of female participants relative to the total number of participants (females + males).

***Order of administration***. *Order of administration* (100%) denoted the position of implicit and explicit measures in the procedure of a study. Relevant information was retrieved from the design, chronologic descriptions, or explicit statements about the order of the instruments. We used four categories: “PSE/TAT -> explicit measure,” “explicit measure -> PSE/TAT,” “not reported/not inferable,” and “varying order.”

***Information source***. *Information source* (Rosenthal, 1991; 100%) was a study's mode of publication, with four categories: “book/chapter,” “dissertation/doctoral-/master's/diploma-/bachelor's thesis,” “journal” and “unpublished.” “Book/chapter” comprised book chapters or books based on one data set. “Journal” comprised papers in which a journal name was given. “Dissertation/doctoral-/master's/diploma-/bachelor's thesis” comprised papers marked as such, even if unpublished. “Unpublished” referred exclusively to unpublished data sets which were not or not yet handed in as theses. There were no missing cases. Relevant information usually was retrieved directly from the text. In all other cases it was found, for instance, in the PsycINFO reference.

***Year***. *Year* (*r* = 1.00) assessed the temporal context of a study. For journal articles or books it was the year of publication. For theses it could also be the year of submission for correction. Except for unpublished data, there were no missing cases. Year was usually reported in the text. If missing, it was coded from other information (e.g., the PsycINFO reference).

### Exclusions

During coding, there were 30 exclusions^5^. Nine papers reported no usable statistics on the implicit-explicit relationship. Four papers were not empirical and no data could be retrieved. Six papers did not treat our topic. Three papers did not use an empirical coding system for the implicit measure. Two studies were reviews themselves (Fineman, 1977; Spangler, 1992), and some of the studies included there were already in our database. Two studies aggregated scales or formed difference scores in the motive measures in a way that did not permit classification of motive domain. Some reasons for exclusion came up only once, such as an implicit motive measure other than the PSE/TAT, results being reported graphically without numerical data, one study being written in Japanese, and one reporting correlations in a highly selective way.

### Statistical methods

We investigated the correlation of implicit and explicit motive measures in a random-effects-model meta-analysis of correlations according to Hunter and Schmidt (2004). The moderator candidates *motive domain, implicit format, explicit construct, order of administration* and *information source* were nominal-scaled; the moderator candidates *age, gender* and *year* were interval-scaled.

Data analyses were run in Systat 12.2 and were based for the most part on the technique proposed by Hunter and Schmidt (2004), which requires no z-transformation for correlations and permits correction for *N* and unreliability in the implicit and explicit measure. In addition, we supplemented this approach with some data-analytic procedures taken from Hofmann et al. (2005), which are described below.

The meta-analytic method of Hunter and Schmidt (2004) is a random effects analysis, based on the assumption that population parameter values may vary in different studies. Therefore, this variance is estimated, in contrast to models using fixed effects where the same population value with a standard deviation of zero is assumed for every study (Hunter and Schmidt, 2004). In a similar vein, Roberts et al. (2007) note that fixed effects models often erroneously detect significant moderators, because Type-I error can increase strongly.

#### Effect size measure

All included correlations except one were uncorrected Pearson correlations. The small downward bias associated with using uncorrected correlations is typically smaller than the upward bias incurred by a Fisher *z*-transformation (Hunter and Schmidt, 1990). One Spearman's *rho* was retained and treated like the other correlations, because *rho* is equivalent to a Pearson correlation between ranks with identical sampling error variance (Hunter and Schmidt, 2004).

#### Correction of study artifacts

We corrected for three study artifacts: (1) sampling error and (2) measurement error in the implicit and (3) explicit measure, respectively. Sampling error is an unsystematic error source mainly determined by *N* (Hunter and Schmidt, 2004). Because *N*s associated with single correlations ranged from 22 to 370, a correction seemed necessary. Correcting for measurement error as our indicator of study quality seemed necessary, too, because it is the only systematic artifact present in every study in a meta-analysis that could lead to an attenuation of the actual correlation (Hunter and Schmidt, 2004). According to Hunter and Schmidt (2004), if 75% or more of the variance in study correlations is accounted for by correctable artifacts, the remaining variance can be attributed to other artifacts not corrected for as well (such as reporting or transcription errors). In this case, substantive moderators are unlikely to be present and would not be tested (75% rule). Like Hofmann et al. (2005), we made no other artifact corrections to avoid departing too much from the original data.

#### Reliability estimates for single correlations

In general, to make measurement error correction possible, missing values for reliability in the implicit and explicit measure were substituted by estimates (cf. Hofmann et al., 2005). In the present sample, reliability estimates were difficult to make. Often no or incomplete reliability information was provided. Moreover, many different indices were reported, seven for the implicit and nine for the explicit measure. However, Hunter and Schmidt (2004) stress that even with suboptimal reliability estimates a correction for measurement error produces results which may be conservative but still are far more accurate than without correction.

For the implicit measure, wherever possible we used reports of inter-rater reliability as reliability information (86 cases); else we used reports on the agreement on practice materials. This procedure yielded reliabilities for 135 cases (80.84% of all cases). Because internal consistency coefficients are inappropriate for the PSE/TAT (Schultheiss and Pang, 2007), percentage agreement was most frequently reported as a reliability estimate (67 cases). This posed a serious problem, because percentage values are not based on correlations and thus not comparable to the internal consistency measures typically reported for explicit tests. Likewise, coefficient kappa, a chance-corrected measure (Bortz and Döring, 2002), which was also reported in some cases, is not directly comparable to either percentage-based or correlation-based reliability estimates.

To achieve comparability with the explicit measure, we therefore deleted percentage-agreement and kappa values and estimated reliability in the form of correlations. Coefficients like Pearson's *r*, Spearman's *rho* or Kendalls *Tau-b* were provided for 54 cases (32.34% of all cases). Except for *r* and *rho* (more than 50% of given correlations), these coefficients are not completely identical measures, but computing means among such correlation-based reliability estimates seemed far less problematic than between correlations and other coefficients. We replaced missing values for the remaining cases by the mean reliability (*M* = 0.857; *SD* = 0.085).

For the explicit measure, reliability was reported 81 times (48.50%). We deleted information on five cases, because the type of reliability estimate was not clearly identified. In this case, too, we deleted the reliability for 13 cases using percentage agreement or Cohen's Kappa. The remaining cases were classified as “not reported clearly” or “no information given.” In the case of the frequently used PRF, we replaced missing values with the reliabilities provided in the manual (Jackson, 1999). For other frequently used measures, we replaced missing values with the best estimators from other studies by, for instance, transcribing the reliability value reported there for the same measure into the missing case in question. Thus, eventually all values represented coefficients of equivalence like Cronbach's Alpha, odd-even, split-half, or Kuder-Richardson 20, and we were therefore able to calculate the mean reliability for explicit measures (*M* = 0.747; *SD* = 0.097; *n* = 95). Finally we replaced missing values by mean reliability of measure category *for ad-hoc* and standardized measures^4^ separately.

#### Combination of correlations within single studies

Studies reported on average 2.98 correlations, 1.67 coding systems and 1.46 explicit measures. Correlations in meta-analyses should be independent of each other, i.e., based on non-overlapping samples (Rosenthal and DiMatteo, 2001). Therefore, prior to estimating ρ, multiple dependent cases within the same study (i.e., same sample) were combined into one study correlation, either overall or within defined moderator categories, depending on the analysis (cf. Rosenthal and DiMatteo, 2001).

The uncorrected study correlation *r*_study_ was the mean of the single values weighted by sample size (Hofmann et al., 2005). Single correlations [*r*_ui(single)_] were weighted by corresponding sample size, added up, and divided by the sum of weights. Weighting was necessary, because within studies the same subjects were sometimes administered multiple measures, but their number fluctuated from measure to measure. By weighting we could use the overall number of subjects as final *N*, because the rare single correlations based on a subsample only thereby received less weight in the study correlation.

For computing corrected study correlations, we first corrected the single correlations by individually dividing them by the product of the square roots of the reliabilities of the implicit and explicit measure. The corrected study correlation *r*_ci_ was then computed analogously to *r*_study_, but the weighting *w*_i_ of corrected correlations included *A*^2^_i_ in addition to *N*_i_:

$$r_{\text{ci}}=\frac{\sum w_{\text{i}}*{\text{r}}_{\text{ci}}(\text{single})}{\sum w_{\text{i}}};$$

where w_i_ = N_i_ * A^2^_i_; with A^2^_i_ = (r_ui(single)_/r_ci(single)_)^2^.

The *squared artifact multiplier A*^2^_i_, represented by the squared ratio of the uncorrected to the corrected single correlation (Hunter and Schmidt, 2004; Hofmann et al., 2005), reduces the weight of correlations as their artifact attenuation gets more extreme, thereby assigning studies with more information more weight.

Computation of study correlations was repeated for every hypothesis before continuing with the steps below, because each time only cases with data on a given moderator candidate or category could be included in the respective analysis. Therefore, number of study correlations varies for different analyses. For instance, for analyses concerning specific motive domains (e.g., *n* Achievement), all motive-specific correlations within a study were aggregated separately and treated as respective study correlations, while cases or whole studies featuring no data on the respective domain (e.g., those restricted to *n* Affiliation and/or *n* Power) were not included in the corresponding analysis.

#### Computations on study correlations

First, we estimated the mean population correlation ρ using study correlations corrected for sampling and measurement error. By weighting study correlations by *N* and reliability of the measures, our computing method across studies was analogous to aggregating corrected single correlations into study correlations:

$$\rho ={\bar{r}}_{\text{c}}=\frac{\sum w_{\text{i}}*{\text{r}}_{\text{ci}}}{\sum w_{\text{i}}}$$

Next, we estimated the observed variance of corrected study correlations var(r_c_):

$$\text{var}(r_{\text{c}})=\frac{\sum w_{\text{i}}*{\left({\text{r}}_{\text{ci}}\text{-}{\bar{r}}_{\text{c}}\right)}^{2}}{\sum w_{\text{i}}}$$

As var(*r*_c_) is also affected by sampling error variance var(e), we estimated the proportion of the latter in var(*r*_c_). Therefore we had to estimate sampling error variance *v*_i_ in every single study i:

$$v_{\text{i}}=\frac{{\left(1-{\bar{r}}_{\text{u}}^{2}\right)}^{2}}{\left({\text{N}}_{\text{i}}-1\right)*A_{\text{i}}^{2}};$$

where *r*_u_ = population mean of study correlations corrected for sampling error only

*v*_i_ was used to estimate var(e):

$$\text{var}(\text{e})=\frac{\sum w_{\text{i}}*v_{\text{i}}}{\sum w_{\text{i}}}$$

To determine the true variance of the population coefficient var(ρ), we then subtracted var(e) from var(*r*_c_). From the square root of var(ρ), we estimated the standard deviation of the population coefficient *SD*_ρ_.

We made our moderator analyses dependent on Hunter and Schmidt's (2004) 75% criterion (see above). For this purpose, we computed V%, defined as var(e) divided by var(*r*_c_). (Formulas and computation methods taken from Hofmann et al., 2005; cf. Hunter and Schmidt, 2004)

### Use of confidence intervals

To estimate if a correlation is different from zero, we constructed two-tailed 95% CIs around ρ according to standard formulas (cf. Collins et al., 2004; e.g., Bortz, 2005). The determination of CIs was also important for comparing the results of our meta-analysis to those of Spangler (1992). In addition, moderators are more effectively identified if specified a priori and tested with CIs around subgroup means than if the existence of moderators is examined a posteriori by heterogeneity tests (Hunter and Schmidt, 2004). For CIs used in testing our main hypotheses and nominal-scaled moderator candidates, we needed an approximation of the standard error of ρ:

$$SE_{\rho }=\frac{SD_{\text{rc}}}{\sqrt{k}};$$

where *k* = number of studies (Hunter and Schmidt, 2004).

*SD*_rc_ is not *SD*_ρ_, but the standard deviation of individually corrected study correlations.

### Moderator analyses

For nominal-scaled variables, we divided correlations according to previously defined categories, aggregated them separately, and placed CIs around category means (Hunter and Schmidt, 2004). If the CIs of two categories did not overlap, we interpreted this as evidence for a moderator effect. We excluded categories with less than five study correlations from our analyses (cf. Hofmann et al., 2005).

For interval-scaled moderator candidates, the strength of their relationship to ρ was estimated with correlations and 95% CIs. In these analyses, we used all studies reporting data for the respective candidate variable, including study correlations corrected for sampling- and measurement error and the variable itself. Then we ran a meta-analysis as described above. Finally we computed Cor(ρ, y), the correlation of moderator candidate y with ρ:

$$\text{Cor}(\rho ,\text{y})=\frac{\text{Cor}(\text{r},\text{ y})}{\sqrt{\text{Var}(\rho )/\text{var}(\text{r})}}$$

where Cor(*r*, y) = correlation of the moderator candidate with the size of the correlations in the single studies (Hunter and Schmidt, 2004).

## Results

### Data preparation

#### Deleted cases and treatment of outliers

Prior to analysis, we deleted seven correlations because of incomplete conceptual fit. For instance, in one study a helping motive was defined as an avoidant power motive (1 correlation) while another study reported correlations which could not be classified as between similarly oriented measures (6 correlations; see inclusion criterion 2). Because we retained conceptually adequate correlations from the two studies involved (Fisch and Schmalt, 1970; Blickle, 1998), this did not lead to a loss of subjects. In the end, 167 single correlations remained. We ran all analyses with and without outliers identified in a box-plot diagram. Because outliers did not affect conclusions from our analyses substantially, we only report results with outliers included.

### Descriptive statistics

After data collection and analysis, 46 papers and three unpublished data sets remained. The papers covered a time range from 1955 to 2007 and the unpublished data were extracted from current projects in 2009. As Table 2 shows, *N*s for the overall correlation and for *n* Achievement and *n* Affiliation were larger than and *N* for *n* Power approximately equal to the 2785 subjects Spangler (1992) included in his meta-analysis of *n* Achievement.

**Table 2:** **Sample size, number of single correlations, and number of studies^a^ from which data were extracted**.

**Motive domain**	**Sample size**	**Number of single correlations**	**Number of studies**
Overall	6151	167	56
Affiliation	4060	52	36
Achievement	5212	87	48
Power	2601	28	21

*“Number of correlations” for the overall relationship is the sum of single correlations from the motive domains. A study could investigate several motives simultaneously, contributing to “sample size” and “number of studies” for more than one motive domain*.

*^a^Studies are independent samples, not papers*.

Mean age of subjects ranged from 16.62 to 74.73 years. Combining quantitative data for sex of subjects across all studies (4 missing cases) showed that 49.26% were female and 50.74% male. In 47 studies students served as subjects, including 23 studies with psychology majors or students taking psychology courses. One study had a predominantly clinical sample, six studies used non-student and non-clinical samples, and in two studies sample type information was missing.

Table 3 shows all studies with *N* and sample type. Study correlations are listed instead of 167 single correlations, because often multiple correlations were based on one sample, in one case even 14 (King, 1995). Study correlations were calculated by combining the pertinent single correlations while considering sampling error. For better comparability we report explicit constructs instead of the names of the 48 explicit measures used in the papers. Study correlations ranged from −0.210 to 0.610.

**Table 3:** **Overview over accepted studies including sample size and sample type, explicit constructs and study correlations**.

**Number**	**Report**	***N***	**Sample type**	**Explicit construct**	***r***
1.	Biernat, 1989	72	Psychology students	Motives	−0.070
2.	Blickle, 1998	370	Other students	Motives	0.000
3.	Blumenthal et al., 1985	40	Psychology students	Motives	0.197
4.	Brunstein and Hoyer, 2002	88	Other students	Motives	0.080
5.	Brunstein et al., 1995	60	Other students	Goals	−0.040
6.	Brunstein and Maier, 2005, S1	96	Other students	Motives	0.050
7.	Brunstein and Maier, 2005, S2	96	Other students	Motives	0.020
8.	Brunstein and Maier, 2005, S3	96	Other students	Motives	0.120
9.	Craig, 1996	46	Other students	Motives	0.260
10.	Craig et al., 1994	38	Other students	Motives	0.020
11.	DeCharms et al., 1955	78	Not reported	Motives	0.230
12.	Eig, 1996	77	Community-Sample	Motives	−0.060
13.	Emmons and McAdams, 1991	72	Psychology students	Goals, motives	0.195
14.	Fisch and Schmalt, 1970	34	Psychology students	Motives	−0.105
15.	Fleeson, 1992	58	Psychology students	Goals	0.013
16.	Gelbort and Winer, 1985	60	Psychology students	FS	−0.080
17.	Hofer et al., 2006a	177	Other students	Motives	0.080
18.	Hofer and Chasiotis, 2003^a^	120	Community-Sample	Goals	0.170
19.	Hofer et al., 2006b	319	Not reported^c^	Motives	0.025
20.	Holmes and Tyler, 1968	72	Psychology students	Motives, goals	0.100
21.	Jacob, 1996	97	Community-Sample	Motives	0.140
22.	Karabenick, 1977, S1	98	Psychology students	Motives, sensitivity to rejection	0.200
23.	Karabenick, 1977, S2	33	Psychology students	Motives, sensitivity to rejection	0.013
24.	King, 1995	101	Psychology students	Motives, goals, wishes	0.032
25.	Koestner et al., 1989	81	Predominantly clinic patients	Motives	0.093
26.	Kwon et al., 2001	50	Psychology students	Soc., Aut.	0.375
27.	Langan-Fox, 1991, S1	93	Other students	Motives	0.055
28.	Langan-Fox, 1991, S2	110	Other students	Motives	0.100
29.	Langens, 2007, S1	72	Other students	Motives	0.085
30.	Langens, 2007, S2	147	Other students	Goals	0.035
31.	McClelland et al., 1989a^b^, S1	55	Other students	Motives	−0.063
32.	McClelland et al., 1989a^b^, S2	54	Other students	Motives	0.040
33.	McClelland et al., 1998^a^	147	Community-sample	Motives	0.180
34.	Metz-Göckel and Leffelsend, 2001	156	Other students	FF, HS, Motives, goals	0.052
35.	Nakash and Brody, 2006	127	Psychology students	Motives	−0.027
36.	Niitamo, 1999, S1	103	Psychology students	Motives	0.028
37.	Niitamo, 1999, S2	140	Community-Sample	Motives	0.080
38.	Niitamo, 1999, S3	82	Community-sample	Motives	−0.057
39.	Pang, 2006, S1	96	Psychology students	FF, HS	−0.015
40.	Pang, 2006, S2	86	Psychology students	FF, HS	−0.040
41.	Pang and Schultheiss, 2005	323	Other students	Motives	0.043
42.	Schroth, 1985	90	Psychology students	Motives	0.473
43.	Schroth, 1987	120	Psychology students	Motives	0.070
44.	Schultheiss and Brunstein, 2001^b^	195	Other students	Motives	0.077
45.	Sherwood, 1966, S1	67	Psychology students	Motives	0.375
46.	Sherwood, 1966, S2	80	Psychology students	Motives	0.380
47.	Sinha and Prasad, 1978	260	Other students	Motives	0.610
48.	Stanton and Schultheiss, 2007	49	Other students	Motives	−0.210
49.	Thrash and Elliot, 2002	167	Psychology students	FF, Motives, Goals	0.166
50.	Thrash et al., 2007	203	Psychology students	Motives	0.046
51.	Woike, 1995	195	Psychology students	Motives	0.055
52.	Wotruba and Price, 1975	65	Other students	Motives	0.088
53.	Yamauchi and Doi, 1977	77	Psychology students	Motives, goals	0.056
54.	Rösch and Schultheiss, n.d.^d^	80	Other students	Motives	−0.067
55.	Kordik and Schultheiss, n.d.^e^	87	Other students	Motives	0.193
56.	Kordik and Schultheiss, n.d.^f^	96	Other students	Motives	0.193

*The insertion of study identifiers (like S1, S2 and so on) indicates separate samples within a paper, but does not necessarily denote that these data sets were described as separate studies or numbered identically there. The term “psychology students” refers to everyone enrolled in psychology courses. Number, study number; N, sample size; r, study correlation (Aggregation of single correlations corrected for sampling error); explicit construct, construct on which the explicit measure was based, in the case of several measures with different constructs more than one entry per study was made; Aut., autonomy; FF, fear of failure; FS, fear of success, HS, hope for success; Soc., sociotropy*.

a *Flagged: Study reported correlations selectively*.

b *Study also covered results from other publications in the literature list: Number 31 and 32 covered McClelland et al. (1989b), Number 44 covered Schultheiss and Brunstein (1999) and Brunstein et al. (1998)*.

c *The respective study investigated a cross-cultural sample, a feature that was coded in another variable^4^*.

d ^*^Rösch, A. G., and schultheiss, O. C. (n.d.). *The standardized and motivated facial expressions of emotion (SMoFEE) stimulus pool: validity, reliability and intensity ratings*. Friedrich-Alexander University Erlangen-Nürnberg, Erlangen.

e ^*^Kordik, A., and Schultheiss, O. C. (n.d.). The implicit achievement motive: nonverbal indicators of affect I. Unpublished manuscript. Friedrich-Alexander University Erlangen-Nürnberg, Erlangen.

f ^*^Kordik, A., and Schultheiss, O. C. (n.d.). The implicit achievement motive: nonverbal indicators of affect II. Unpublished manuscript. Friedrich-Alexander University Erlangen-Nürnberg, Erlangen.

### Hypothesis testing

#### Main hypothesis 1: overall correlation

The top third of Table 4 shows the main parameters for four meta-analyses. Investigating moderator candidates was justified, because only around 39% of the variance was due to sampling error. Table 4 shows that, as hypothesized, there was a small, positive correlation between implicit and explicit motive measures. The 95% CI did not include zero, so main hypothesis 1 was confirmed.

**Table 4:** **Meta-analytic results for overall relationship of implicit and explicit motive measures and for single motives as well as statistics for uncorrected single correlations**.

	**ρ**	***SD*_ρ_**	**95% CI**	**V%**	***K***	***N***
**CORRELATIONS CORRECTED FOR SAMPLING- AND MEASUREMENT ERROR^a^**						
Overall	0.130	0.162	(0.077, 0.183)	39.16	54	5884
Affiliation	0.116	0.109	(0.050, 0.182)	53.15	34	3832
Achievement	0.139	0.175	(0.080, 0.198)	33.74	47	5092
Power	0.038	0.134	(−0.055, 0.131)	43.68	20	2282
**CORRELATIONS CORRECTED FOR SAMPLING ERROR ONLY^a^**						
Overall	0.095	0.112	(0.056, 0.134)	38.81	54	5884
Affiliation	0.092	0.087	(0.040, 0.144)	53.93	35	3913
Achievement	0.110	0.131	(0.066, 0.154)	34.67	47	5092
Power	0.026	0.099	(−0.046, 0.098)	45.45	21	2601
**DESCRIPTIVE STATISTICS FOR UNCORRECTED SINGLE CORRELATIONS**						
	***M***	***SD***	**95% CI**		***K*^c^**	
Overall	0.073	0.161	(0.048, 0.098)		165	
Affiliation	0.081	0.175	(0.032, 0.130)		51	
Achievement	0.073	0.155	(0.039, 0.106)		86	
Power	0.059	0.155	(−0.001, 0.119)		28	

*ρ, mean population correlation; SD_ρ_, estimated standard deviation of population correlations; CI, confidence interval; V%, percentage of variance accounted for by sampling error; K, number of independent study correlations; N, total sample size; M, arithmetic mean of single correlations; SD, standard deviation*.

a *K is slightly lower than in the overall statistic in Table 2 due to the exclusion of two flagged cases and some zero results in our computations*.

b *K is slightly lower than in the overall statistic in Table 2 due to the exclusion of two flagged cases*.

c *For the non-meta-analytic computations, K is the number of single correlations*.

#### Main hypothesis 2: n Achievement

We expected a small positive correlation for *n* Achievement, which would replicate the findings of Spangler's (1992) meta-analysis. We obtained the highest ρ of all motive domains for *n* Achievement (Table 4). The 95% CI did not include zero, but covered the effect size found by Spangler (1992). Main hypothesis 2 was confirmed.

#### Main hypothesis 3: n Affiliation

We expected a small, positive correlation for *n* Affiliation. Table 4 shows a value for *n* Affiliation similar to the overall correlation. As the 95% CI did not include zero, main hypothesis 3 was confirmed.

#### Main hypothesis 4: n Power

For *n* Power, we expected a coefficient which would not differ from zero. Here, ρ was clearly smaller than that for the other motives and the overall analysis (Table 4). The 95% CI for *n* Power included zero. Thus, main hypothesis 4 was confirmed.

#### Results for study correlations corrected for sampling error only

The middle third of Table 4 shows the results for a “bare-bones“ meta-analysis (Hunter and Schmidt, 2004) in which only sampling error was corrected and measurement error was not considered. We report these results in addition to our main analyses, because, as stated above, estimation of reliabilities was difficult in our database and so inaccurate estimates cannot be ruled out. Except for using study correlations without correction for measurement error and omitting the squared artifact multiplier, computations were like the ones described above. As the CIs show (Table 4), conclusions for all four main hypotheses were the same as in the main analyses even if measurement error was not considered.

#### Moderator analyses

We assumed the presence of moderators, because V% never reached 75% when we tested main hypotheses 1–4. Because CIs for all motives overlapped, moderator candidates were tested for the overall relationship of implicit and explicit motive measures. Table 5 shows the results for nominal-scaled, Table 6 for interval-scaled moderator candidates.

**Table 5:** **Results of moderator analyses for nominal scaled variables**.

**Variable**	**ρ**	***SD*_ρ_**	**95% CI**	**V%**	***K***	***N***
**IMPLICIT FORMAT**						
Paper and Pencil	0.136	0.189	(0.070, 0.202)	32.15	39	4439
PC	0.081	0.140	–	50.16	4	312
Oral	0.248	0	–	100	4	268
**EXPLICIT CONSTRUCT**						
Motives	0.135	0.175	(0.077, 0.193)	35.24	48	5364
Goals	0.094	0	(0.002, 0.186)	100	9	910
Wishes^a^	–	–	–	–	1	85
**ORDER OF ADMINISTRATION**						
Implicit measure first	0.149	0.187	(0.084, 0.214)	32.38	41	4198
Explicit measure first	0.086	0.027	(−0.074, 0.246)	95.85	7	798
Varying order	0.025	0	–	100	2	180
**INFORMATION SOURCE**						
Book/chapter	0.283	0	–	–	1	78
Dissertation	0.043	0	(−0.055, 0.141)	100	9	785
Journal	0.144	0.182	(0.033, 0.255)	33.32	41	4758
Unpublished	0.138	0.071	–	77.08	3	263

*Because there were no non-overlapping confidence intervals for any variable, we refrained from inserting subscripts indicating non-overlapping categories; ρ, mean population correlation; SD_ρ_, estimated standard deviation of population correlations; CI, confidence interval (not computed for categories with less than five study correlations); V%, percentage variance accounted for by sampling error; K, number of independent study correlations; N, total sample size*.

a *Computation of the population mean was not possible due to a weight of zero, the study correlation of the only study found was r = −0.006*.

**Table 6:** **Correlation of quantitative moderator candidates with the population coefficient**.

	**ρ**	**Var(ρ**)	**Cor(*r_c_*, y)**	**Cor(ρ**, y)	**95% CI**	***K***	***N***
Age	0.107	0.031	−0.009	−0.011	(−0.345, 0.325)	34	4000
Gender	0.133	0.029	−0.263	−0.329	(−0.050, −0.550)	50	5340
Year	0.130	0.027	−0.402	−0.511	(−0.685, −0.270)	51	5621

*ρ, mean population correlation; var(ρ), variance of population correlations; Cor(r_c_, y), correlation of the moderator candidate with the correlations in the single studies; Cor(ρ, y), correlation of moderator candidate y with the population coefficient ρ; CI, confidence interval around Cor(ρ, y); K, number of independent study correlations; N, total sample size*.

***Characteristics of the implicit measure***. Analyses for data collection format of the PSE could not be conducted due to the small number of studies reporting PC or oral administration. In addition, in many cases the format could not be inferred (15 correlations) and paper-and-pencil-studies (130 correlations) were clearly preponderant. Whether characteristics of the implicit measure influence the relationship was therefore not testable (Additional hypothesis 1.1).

***Characteristics of the explicit measure***. We divided the variable *explicit construct* into self-reported motives, wishes, and goals. While wishes could not be tested, ρ was lower for goals than for motives, but overlapping CIs indicated no difference. Additional hypothesis 2.1 was not supported, but could not be tested completely. Characteristics of the explicit measure had no detectable influence on the relationship.

**Figure 2:**
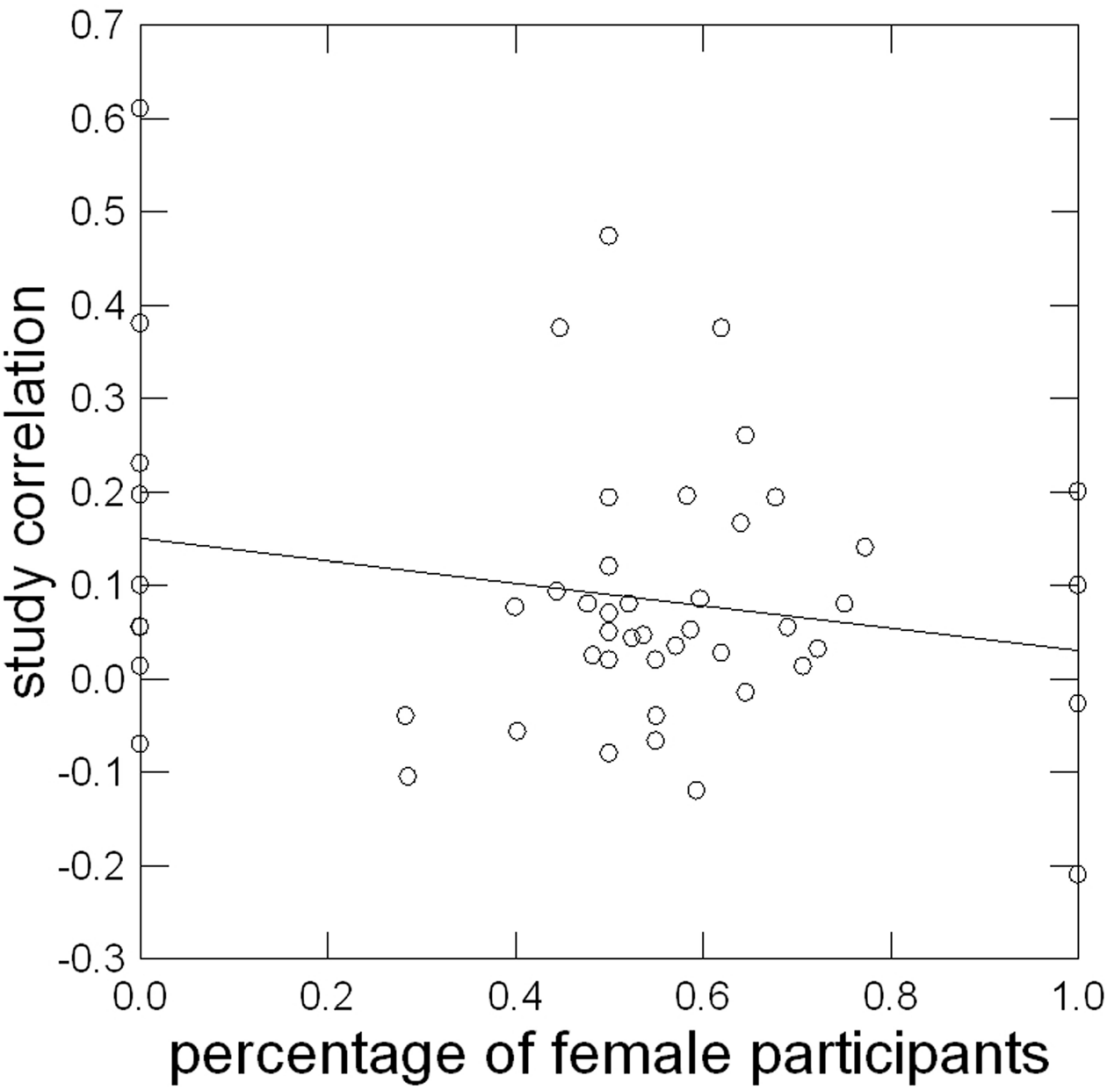
**Linear relationship of percentage of female participants in a sample with study correlation corrected for sampling error**.

***Characteristics of the sample***. Figure 2 shows the relationship of percentage of female participants in a sample with the study correlation. Contrary to our assumption, it was slightly different from zero (see Table 6) and negative: The higher the proportion of women in a sample, the lower the correlation of implicit and explicit motive measures. Additional hypothesis 3.1 concerning gender was not supported.

As expected, age and the relationship of implicit and explicit motive measures were uncorrelated. The CI around Cor(ρ, y) included zero. Additional hypothesis 3.2 was confirmed.

***Characteristics of study design***. Investigating order of administration, we used the categories “implicit measure first,” “explicit measure first,” and “varying order.” While the last category could not be examined, ρ for “implicit measure first” was nominally higher than that for “explicit measure first,” but overlapping CIs indicated no difference. However, the CI for explicit measure first included zero while the one for implicit measure first did not. Additional hypothesis 4.1 was partially supported.

***Origin of studies***. Within *information source*, neither books or book chapters nor unpublished data sets could be examined due to the small number of studies. As expected, ρ for journals was different from zero and visibly higher than the one for dissertations, which did not differ from zero. Because of overlapping CIs, a difference could not be detected. Additional hypothesis 5.1 was partially supported.

**Figure 3:**
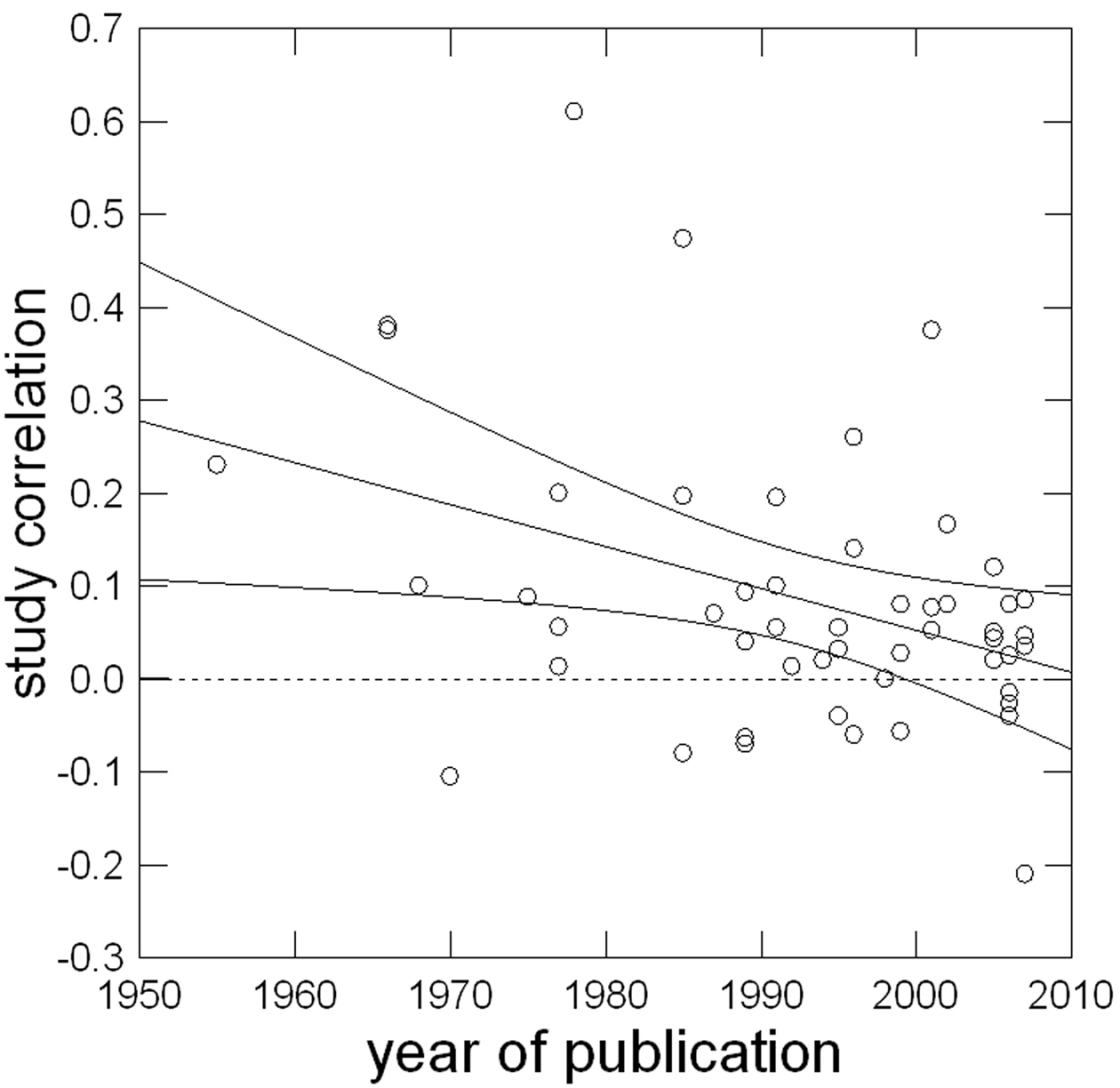
**Linear relationship of year of publication with study correlation corrected for sampling error**.

Contrary to expectations, publication year was negatively related to the relationship, as Figure 3 shows. The CI around Cor(ρ, y) did not include zero. Additional hypothesis 5.2 was not supported; the opposite was true. We added a 95% CI to Figure 3 to determine the year by which the correlation was no longer different from zero. As can be seen in the plot, the CI includes zero approximately by the year 2000. Follow-up analyses indicated that the overall negative correlation between study year and implicit-explicit correlations did not differ by motive domain.

### Additional analyses

#### Search for a possible publication bias

**Figure 4:**
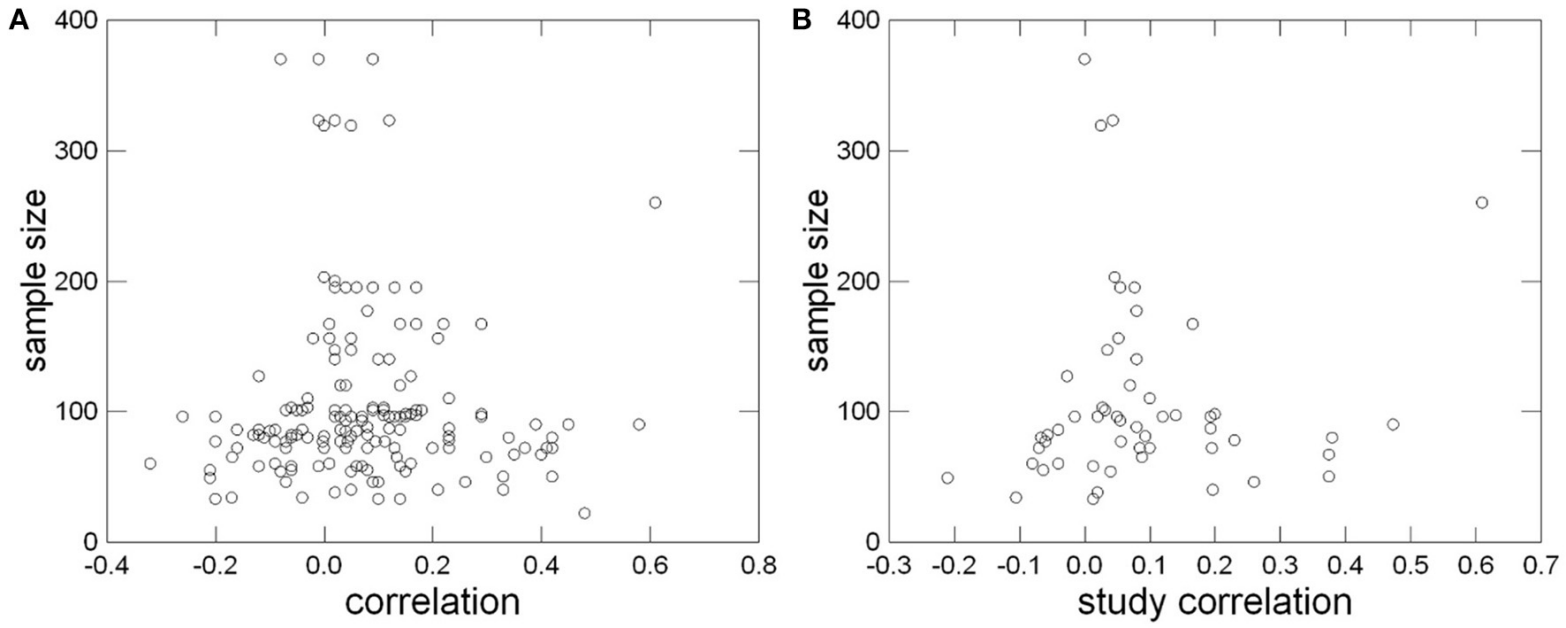
**“Funnel-Plot” of single correlations (A) and of study correlations corrected for sampling error (B) for identification of a publication bias**.

A publication bias can result in overestimation of effect sizes. Like Hofmann et al. (2005), we used a graphical method. A funnel plot detects a publication bias by showing the absence of small effects for studies with small *N*s, because such effects would not become significant. Regardless of *N*, the plots in Figure 4 feature no obvious absence of correlations close to zero. Usually the variance of the correlations in small studies was substantially larger than in studies with large samples. In large samples with more than 300 subjects, values are centered closely around 0.00. The resulting impression of an inverted funnel is desired for a funnel-plot which shows no publication bias (Hunter and Schmidt, 2004; Roberts et al., 2007). But while our funnel plot suggests that a publication bias was unlikely across the entire publication time span of the included studies, in light of the negative association between publication year and the relationship we cannot rule out that a publication bias has existed at some point in implicit motive research.

#### Results for untransformed, uncorrected correlations

We also ran some significance tests to further corroborate our main results. Roberts et al. (2007) mention that even in single studies significance is dependent on *N* in such a way that large studies report small effects as significant, while medium up to large effect sizes are required for the same result in smaller samples. We are aware of this and thus report the following tests as additional analyses only.

The bottom third of Table 4 shows descriptive statistics for uncorrected, untransformed correlations with the two flagged cases excluded. Even if we had done no meta-analysis, but instead had weighted every correlation equally, main hypotheses 1–4 would have been confirmed. A *t*-test for a difference of the overall correlation from zero showed that it was positive and significantly different from zero, *t*_(164)_ = 5.824, *p* < 0.001 (main hypothesis 1). The same was true for *n* Achievement *t*_(85)_ = 4.345, *p* < 0.001, and *n* Affiliation, *t*_(50)_ = 3.297, *p* = 0.002, but not for *n* Power, *t*_(27)_ = 2.011, *p* = 0.054 (main hypotheses 2–4). These conclusions did not change when we either included the flagged studies, or excluded outliers, or excluded outliers and flagged studies simultaneously.

## Discussion

We tested four main hypotheses and explored seven additional hypotheses about the relationships between implicit and explicit motive measures and their moderators. Most importantly, in support for our first main hypothesis, we found a positive, but small correlation between implicit and explicit motive measures in general. We also found support for our second and third main hypotheses. The specific correlation between implicit and explicit *n* Achievement measures was small, but positive, and included in its CI the effect size originally reported by Spangler (1992). Thus, despite only partial overlap in the studies included in his and our meta-analysis, we were able to replicate the effect size he reported. We also documented a similar small, positive correlation for *n* Affiliation, with a CI that overlaps with the one for *n* Achievement. In contrast, and in support of main hypothesis 4, the correlation for *n* Power could not be differentiated from 0. While interpreting observed differences in the correlations for the three motives in terms of social desirability may be consistent with McClelland's (1987) original conjecture, other factors cannot be ruled out. Note, however, that because the CIs of all three motives overlapped, we cannot conclude from our findings that effect sizes for the three motive domains differ significantly or substantially.

While these findings may suggest to some that after all, there *is* some convergence between implicit and explicit motive measures and that this may be due to people having some limited introspective access to their motivational needs, we think that such an interpretation would be premature for the following reasons. First, even after correcting for sample error and measure unreliability, the overlap between implicit and explicit motive measures was modest—1.7% of shared variance. Without corrections, the variance overlap was only 0.5%. Thus, the level of convergence is minuscule and in the range of what Cyders and Coskunpinar (2011) referred to as evidence for independent constructs in their meta-analysis of measures of impulsivity. In line with the theorizing presented in the introduction, the idea of neurobiologically distinct motivational systems, one implicit and guided by affect, the other explicit and tied to the self-concept seems the most plausible explanation for our results (see Hall et al., 2010).

Second, what holds for correlational studies in general also holds for meta-analytic estimates of associations between variables: correlations do not imply causality. In other words, the small, positive correlations we obtained in our overall analysis and also for the specific domains of achievement and affiliation cannot be interpreted to mean that higher levels of implicit motivation somehow also raise a person's explicit sense of motivation (or vice versa). Other factors, perhaps even unrelated to motivation proper (such as time of day of testing; general effects of positive or negative emotionality; etc.), may simultaneously influence both implicit and explicit measures, rendering their slight positive correlation mostly or entirely spurious. Future research therefore needs to employ experimental designs, testing candidate causal processes that could account for the small, positive correlation between implicit and explicit motive measures as well as for variations in this association (see, for instance, Schultheiss and Strasser, 2012). Such studies should go beyond documenting de-facto independence between implicit and explicit measures and test candidate causal processes through explorations of these measures' causal and predictive validities. In so doing, they should aim to establish empirically and systematically the incentives and outcomes associated with the implicit and the explicit level, respectively. A central step in establishing causal validity on the explicit side would be to empirically derive self-report measures in a manner similar to what has been done for the implicit measures from the very beginning—studying the effect of an aroused motivational need on scale scores and thus illuminating the processes that generate responses captured by the measure. In establishing predictive validity, designs like those already used by some researchers (Brunstein and Hoyer, 2002; Brunstein and Maier, 2005; for an excellent recent attempt, see Brunstein and Schmitt, 2010; cf. DeCharms et al., 1955) could be used and existing work on predictive validity could be integrated meta-analytically as Spangler (1992) did for incentives as well as for outcomes related to *n* Achievement. After a systematic integrative model of causal processes in implicit and explicit motivation as well as their main outcomes has been established, the biopsychological bases of both levels of motivation could be identified by using, for example, fMRI-designs, as has already been attempted in some pioneering research (see Hall et al., 2010).

Aside from our main results, the analysis of the interval-scaled moderator candidates revealed some interesting and unexpected findings. Secular trends in motivation science seem to matter, as we found a strong negative relationship between publication year and correlation of implicit and explicit measures. In fact, this was the strongest effect of the whole analysis. Perhaps motive congruence actually dropped over the past 60 years, but in light of the assumed biological substrates for implicit and explicit motives (Schultheiss, 2007) a drop at that rate seems very implausible on neuroevolutionary grounds. The most likely explanation is that at the beginning of motive research, many researchers aimed at replacing the time-consuming PSE with more “economical” questionnaire measures and selectively published studies that supported the hypothesis that such measures converge with the PSE, while delegating non-supporting studies to the notorious desk drawer. Or perhaps researchers feared getting rejected if they were not able to document convergent validity between self-report and PSE-based motive measures.

The influential articles by McClelland (1980) and McClelland et al. (1989a) changed the situation by firmly establishing the notion that implicit and explicit motive measures represent fundamentally different constructs and predict different outcomes. In the wake of these papers, publication of studies that failed to find a substantial overlap between implicit and explicit motive measures became more acceptable and commonplace. We note that this secular trend would suggest that the small positive correlation between implicit and explicit motive measures we and, before us, Spangler (1992) found may actually be due to the selectively published studies from the early phase of motive research whose effect sizes are typically above the average correlation we report here. In contrast, more recent and less selectively published studies find correlations much closer to zero and thus below our average correlation coefficient. Thus, if our interpretation of the reason for the secular effect size trend is correct, our average meta-analytic findings represent an *overestimation* of the true variance overlap between implicit and explicit motive measures.

Unexpectedly, we found the proportion of female participants in a given study sample to be associated with *lower* congruence between implicit and explicit motives. Since we are the first to observe such an effect in the domain of motivation research, we hesitate to interpret it before further corroborating work has been carried out. If pressed to speculate, however, we see two tentative explanations for this effect. One is based on differential gender socialization, with women growing up to be relatively more attuned to others' needs than their own (e.g., Eagly and Carli, 1981; Gilligan, 1993). This process would make them more prone than men to ascribe motivational needs to themselves on questionnaire measures that do not match their implicit motives. The other explanation is based on the recent study by Schultheiss et al. (2012), who reported that high levels of progesterone impair interhemispheric integration and also found evidence that this process leads to greater incongruence between individuals' implicit motives and explicit goals. Because women experience phases of high progesterone levels (e.g., luteal phase of the menstrual cycle; pregnancy) more frequently than men do, they may also be more vulnerable to its congruence-reducing effects.

In line with McClelland et al.'s (1989a) hypothesis that the degree of overlap between people's implicit and explicit motives does not automatically increase throughout the life course, we failed to find an association between age and motive congruence. From a broader perspective, this finding was to be expected, as many of the goals that people adopt at a given life stage are not chosen based on personal motivational preferences, but represent age-graded demands dictated by society and cultural background (cf. Caspi, 1987). This interpretation suggests that only those individuals may learn to integrate their implicit motives into their explicit view of themselves who have learned to recognize, and emancipate themselves from, such sociocultural demands.

For the nominal-scaled moderator candidates, results are less impressive, mainly because in many instances, there were too few studies representing specific categories to derive meaningful statistics from them. For categories with an acceptable number of studies, we usually found small, yet significant population means like in our main analyses.

While effects of the data collection format of the PSE were not testable due to insufficient data on less frequent categories, testing the influence of the explicit construct did not yield a significant difference between goals and self-reported motives. Maybe both constructs are equally adept at assessing distinct forms of explicit motivation which are both equidistant from implicit motivation (cf. Rawolle et al., 2013). Two of our additional hypotheses for nominal-scaled candidates yielded interesting results, even if the CIs of tested categories overlapped in both cases: First, if the explicit measure preceded the implicit, but not vice versa, the small positive correlation between them disappeared and no longer differed from zero. We speculate that this effect may be due to an asymmetrical *PSE/TAT -> explicit measure* priming effect, but further research is needed to substantiate this notion. Second, while case numbers were too small for testing books, book chapters, or unpublished data sets, journal articles produced results in line with the general finding of small and positive correlations, while the relationship was not different from zero for dissertations. The latter result can be interpreted as support for Rosenthal's (1991) assumption that dissertations tend to produce lower effect sizes than other sources, maybe because some of them are methodically inferior to those that survived the peer-review process in journals or because their findings run counter to prevailing orthodoxy. But once again, because of overlapping CIs, these results need to be viewed with caution.

An important question is how robust the results of the meta-analysis were. First, a publication bias was unlikely. For the domain of motive congruence, there is no reason to hold back on publishing non-significant results, because this would confirm general theory since the 1980ies instead of challenging it. And even if a study had significant results, we would expect them to be published as well, because we think it is unlikely that papers are rejected for finding the occasional significant correlation. Moreover, such findings are interesting in their own right, because they may point to moderators of motive congruence. So it was no surprise that a graphic funnel-plot test showed no indications of a publication bias. But while our overall funnel plot analysis suggested that a publication bias is unlikely, we cannot rule out that this finding obscures a secular trend similar to the one we found between publication year and correlation size. Perhaps a publication bias favoring substantial positive correlations was present in the early years of motive research, but vanished with the advent of theories emphasizing the independence between implicit and explicit motivational systems (e.g., McClelland et al., 1989a). Even if this were true, it would not endanger the conclusions drawn for our main hypotheses, as the general funnel plot had the desired shape. Second, every main hypothesis test was based on several thousand subjects, adding to the robustness of results. But above all, the conclusions drawn for our main hypotheses remained the same if outliers were removed, only sampling error was corrected or even if a non-meta-analytical approach was chosen ignoring the dependence of some correlations and using conventional significance tests.

### Limitations

One limitation of our literature search was restricting the PsychINFO search to papers with the highest chance of relevance and only using one rater to screen them. We may therefore have missed relevant studies with less informative abstracts, keywords, or title, or papers reporting an implicit-explicit correlation only as an additional analysis. But as we were only interested in a sufficient approximation of the true correlation and as we also had a comprehensive list of key studies to cover less well-known studies, this should not have biased our main results and the conclusions drawn from them.

Another limitation was beyond our control: Moderator analyses were not as conclusive as we had hoped. In many cases there were not enough studies to reveal possible effects or differences. But aside from the a-priori selection of moderator candidates, we had no influence on the frequency of relevant information in the study sample. This problem is therefore not specific to our mode of analysis.

Further, it has to be noted that the literature search terminated five years ago, but given that new findings in this area go in the same direction (see for example Schultheiss et al., 2009; Rawolle et al., 2013) and the large number of already included studies, no substantial changes in the results were to be expected if the most recent studies had been included, too.

A final limitation of our meta-analysis was the weak basis for the correction of measurement error. Vast amounts of reliability data on the implicit measures had to be deleted and replaced by estimates because kappa and percentage agreement measures were not commensurable with variance-based reliability measures. For explicit measures, the reliability values used for analysis were restricted to coefficients of equivalence, not of stability, because none of the studies reported the latter. We concede that, if the required reliability data had been available for all studies and measures, better correction for measurement error might have resulted in less biased effect size measures. Note, however, that our main hypothesis tests with and without corrections for measurement error all converged on the same essential conclusions, which suggests that our findings may not strongly depend on such corrections.

### Future research

To the extent that our results can be generalized to other theories, the robust finding that content coding and self-report measures of motivation do not converge substantially (see also Cyders and Coskunpinar, 2011) could also spell trouble for related theoretical approaches based on Bakan (1966, cf. e.g., Abele and Wojciszke, 2007, for more recent research on this topic) or on self-determination theory (SDT, Deci and Ryan, 2000), which frequently rely on self-report measures. These rely on the premise that introspection provides valid data. Our findings suggest that these measures may only confer an incomplete picture, because they are unlikely to converge with more indirect, implicit measures of their target attributes.

For future reviews and meta-analyses concerning implicit and explicit measures of motivation, a more unitary standard of reporting data would be desirable, especially for measurement reliability, because in the present study much information on measurement error was lost due to incompatible indices used. Most critically, the reporting of PSE reliability should make use of variance-based measures such as intra-class correlation indices (see Shrout and Fleiss, 1979) or Lin's index of concordance (Lin, 1989) in the future.

An important potential moderator, ethnicity/cultural background, could not be tested because of the fact that explicit information on this matter was extremely sparse and using the location of the institutional affiliation of the respective first authors as best estimator proved to be an error-prone and incomplete way of gaining information on this variable, originally named “sample nationality.” Future studies should report exact information on ethnicity/cultural background, or at least on the nationality of the subjects in order to allow clear-cut analyses on this matter.

Another potentially important moderator that we were not able to examine in the present meta-analysis is the degree of commensurability of implicit and explicit motive measures. As Schultheiss et al. (2009) pointed out, PSE measures of implicit motives assess research participants' imagined responses to specific situational cues, using content coding categories derived through experimental motive arousal studies. By contrast, explicit motive measures typically consist of decontextualized items describing general behavioral propensities. They are derived through a-priori assumptions about what constitutes a particular motive and are selected and optimized to conform to criteria of reliability and factorial purity rather than causal sensitivity for the attribute they are supposed to measure. In recent years, some studies have started to address this lack of commensurability by devising self-report measures that either try to match the content-coding systems used for the assessment of specific motives through items designed to specifically represent each content coding category (e.g., Thrash et al., 2007) or by going one step further and presenting such coding-system-matched items coupled to the same picture cues used in the PSE (e.g., Schultheiss et al., 2009). However, despite initial evidence suggestive of higher convergence between such matched measures (Thrash et al., 2007), other studies failed to replicate this observation (Schultheiss et al., 2009, 2011; Ramsay and Pang, 2013). As more studies using commensurable measures of implicit and explicit motives are conducted and published, future meta-analyses can focus on the harvest of these studies and help further determine whether the low positive correlations between implicit and explicit motive measures we report here are an artifact of incommensurable measures or represent a genuine phenomenon.

## Conclusion

To conclude, we replicated Spangler's (1992) small positive correlation for *n* Achievement with a different meta-analytical approach. We extended this result to *n* Affiliation, found a smaller correlation that could not be differentiated from zero for *n* Power, and obtained evidence that the average degree of overlap between implicit and explicit motives may be driven more strongly by older studies than more recent ones. We hope that we have thus helped to resolve the 60 year-old research question about the relationship between PSE and self-report measures of motivational needs.

## Author contributions

Martin G. Köllner conducted all steps of the meta-analysis with exception of compiling the “positive list,” parts of the requests for papers and the subset-coding of a second coder for determining inter-rater reliability. He further conducted all data analyses, prepared figures and tables as well as the manuscript. Oliver C. Schultheiss gave advice on and supervised all phases from the beginning of data collection to data analyses and revised the manuscript. He further compiled the “positive list” as a basis for evaluating our data collection and made some requests for papers during the literature search.

### Conflict of interest statement

The authors declare that the research was conducted in the absence of any commercial or financial relationships that could be construed as a potential conflict of interest.

## Footnotes

^1^The current journal coverage of PsycINFO can be found at: http://www.apa.org/pubs/databases/psycinfo/coverage.aspx

^2^A copy of the complete coding form with all 61 variables assessed is available from the first author.

^3^A list of all studies used in determining reliability is available from the first author.

^4^In addition to the analyses reported here, we also analyzed other potential moderators, such as type of explicit measure (ad-hoc versus standardized), sample type (psychology students, other students, community sample, clinical sample), and number of PSE pictures. However, type of explicit measure did not yield substantial differences and due to the rare occurrence of one or more subcategories for sample type and number of PSE pictures, no meaningful analyses could be performed. We therefore do not report the mentioned variables and analyses here. We also considered testing for moderation by the explicit measures used, but due to diverse and many infrequent categories, no meaningful analyses could be conducted.

^5^A list of studies excluded during coding is available from the first author.

## Supplementary material

List of all terms and term combinations used in the PsycINFO-search.
