# Supplementary material for: Meta-analytic evidence of low convergence between implicit and explicit measures of the needs for achievement, affiliation, and power
Source: Front Psychol. 2014 Aug 8;5:826. doi: 10.3389/fpsyg.2014.00826 (PMC4126572; doi:10.3389/fpsyg.2014.00826)
Supplement: Supplementary file 1 [file DataSheet1.DOCX]

***Supplementary Material***

**Meta-analytic evidence of low convergence between implicit and explicit measures of the needs for achievement, affiliation, and power**

**Martin G. Köllner^1^*, Oliver C. Schultheiss^1^**

^1^Human Motivation and Affective Neuroscience Lab, Institute of Psychology, Department of Psychology and Sport Sciences, Friedrich-Alexander University Erlangen-Nürnberg (FAU), Erlangen, Germany

*** Correspondence:**

Martin G. Köllner

Human Motivation and Affective Neuroscience Lab,

Institute of Psychology,

Department of Psychology and Sport Sciences,

Friedrich-Alexander University (FAU),

Nägelsbachstrasse 49b,

Erlangen, 91052, Germany.

[martin.koellner@fau.de](mailto:martin.koellner@fau.de)

1. **Appendix: List of all terms and term combinations used in the PsycINFO-search**

achievement motivation and congruence

achievement motivation and congruency

achievement motivation and CPI

achievement motivation and explicit

achievement motivation and explicit goal

achievement motivation and explicit goals

achievement motivation and personal goa*

achievement motivation and personality scal*

achievement motivation and PRF

achievement motivation and self-rating

achievement motivation and self-ratings

achievement motivation and self-repor*

achievement motivation CPI

achievement motivation congruence

achievement motivation congruency

achievement motivation explicit goal

achievement motivation explicit goals

achievement motivation personal goal

achievement motivation personal goals

achievement motivation personality scale

achievement motivation personality scales

achievement motivation PRF

achievement motivation self-rating

achievement motivation self-ratings

achievement motivation self-report

achievement motivation self-reports

achievement motive and congruence

achievement motive and congruency

achievement motive and CPI

achievement motive and explicit

achievement motive and explicit goal

achievement motive and explicit goals

achievement motive and personal goal

achievement motive and personal goals

achievement motive and personality scale

achievement motive and personality scales

achievement motive and PRF

achievement motive and self-rating

achievement motive and self-ratings

achievement motive and self-report

achievement motive and self-reports

achievement motive congruence

achievement motive congruency

achievement motive CPI

achievement motive explicit

achievement motive explicit goal

achievement motive explicit goals

achievement motive personal goal

achievement motive personal goals

achievement motive personality scale

achievement motive personality scales

achievement motive PRF

achievement motive self-rating

achievement motive self-ratings

achievement motive self-report

achievement motive self-reports

achievement motives and values

affective and cognitive and motiv* and (congruenc* or correlation or relation or relations or relationship or independence)

affective and cognitive motivation

affiliation motivation and congruence

affiliation motivation and congruency

affiliation motivation and CPI

affiliation motivation and explicit

affiliation motivation and explicit goal

affiliation motivation and explicit goals

affiliation motivation and goal

affiliation motivation and goals

affiliation motivation and personal goal

affiliation motivation and personal goals

affiliation motivation and personality scal*

affiliation motivation and PRF

affiliation motivation and self-rating

affiliation motivation and self-ratings

affiliation motivation and self-report

affiliation motivation and self-reports

affiliation motivation congruence

affiliation motivation congruency

affiliation motivation CPI

affiliation motivation explicit

affiliation motivation explicit goal

affiliation motivation explicit goals

affiliation motivation goal

affiliation motivation goals

affiliation motivation personal goal

affiliation motivation personal goals

affiliation motivation personality scale

affiliation motivation personality scales

affiliation motivation PRF

affiliation motivation self-rating

affiliation motivation self-ratings

affiliation motivation self-report

affiliation motivation self-reports

affiliation motive and congruence

affiliation motive and CPI

affiliation motive and explicit goal

affiliation motive and explicit goals

affiliation motive and personal goal

affiliation motive and personal goals

affiliation motive and personality scale

affiliation motive and personality scales

affiliation motive and PRF

affiliation motive and questionnaire

affiliation motive and self-rating

affiliation motive and self-ratings

affiliation motive and self-report

affiliation motive and self-reports

affiliation motive congruence

affiliation motive congruency

affiliation motive CPI

affiliation motive explicit goal

affiliation motive explicit goals

affiliation motive personal goal

affiliation motive personal goals

affiliation motive personality scale

affiliation motive personality scales

affiliation motive PRF

affiliation motive questionnaire

affiliation motive self-rating

affiliation motive self-ratings

affiliation motive self-report

affiliation motive self-reports

a model of dual attitudes

apperceptive and CPI

apperceptive and personal goal

apperceptive and personal goals

apperceptive and personality scal*

apperceptive and PRF

apperceptive and questionnaire

apperceptive and self-rating

apperceptive and self-ratings

apperceptive and self-report

apperceptive and self-reports

apperceptive CPI

apperceptive explicit goal

apperceptive explicit goals

apperceptive goa*

apperceptive personal goa*

apperceptive personality scal*

apperceptive PRF

apperceptive questionnair*

apperceptive self-ratin*

apperceptive self-repor*

apperceptive method and (CPI or PRF)

apperceptive method and personal goa*

apperceptive method and personality scal*

apperceptive method and questionnair*

apperceptive method and self-ratin*

apperceptive method and self-repor*

apperceptive method CPI

apperceptive method personal goa*

apperceptive method personality scal*

apperceptive method PRF

apperceptive method questionnair*

apperceptive method self-ratin*

apperceptive method self-repor*

apperceptive methods and (CPI or PRF)

apperceptive methods and personal goa*

apperceptive methods and personality scal*

apperceptive methods and questionnair*

apperceptive methods and self-ratin*

apperceptive methods and self-repor*

apperceptive methods CPI

apperceptive methods personal goa*

apperceptive methods personality scal*

apperceptive methods PRF

apperceptive methods questionnair*

apperceptive methods self-ratin*

apperceptive methods self-repor*

Assessing implicit motives with a

congruenc* between explicit motives

congruenc* between implicit and explicit measures

congruenc* between implicit and explicit motives

congruenc* between implicit motives

congruenc* between motives

congruenc* explicit

congruenc* implicit

congruenc* implicit and explicit

congruenc* implicit motives

congruenc* motive

conscious and unconscious motiv*

CPI and apperceptive

CPI and implicit

CPI and implicit motiv*

CPI and motiv*

CPI apperceptive

CPI implicit

CPI implicit motiv*

CPI motiv*

declarative and nondeclarative

declarative and nondeclarative personality

dual-process theor*

explicit achievement

explicit affiliation

explicit and PSE

explicit and TAT

explicit intimacy

explicit motivation

explicit power

explicit TAT

explicit vs implicit motiv*

goal and PSE

goal and motive congruence

goal and motive congruency

goals and motives congruence

goals and motives congruency

goal PSE

goal congruency

goal imagery

goal-motive congruenc*

goal motive congruenc*

goals motiv*

goals motives congruenc*

implicit achievement

implicit affiliation

implicit and explicit achievement

implicit and explicit affiliation

implicit and explicit assessment

implicit and explicit congruenc*

implicit and explicit intimacy

implicit and explicit motiv*

implicit and explicit personality measur*

implicit and explicit power

implicit and self-attributed achievement motiv*

(implicit or unconscious) and (explicit or conscious or self-attributed or self-reported or self-rated) and (motiv* or achievement or affiliation or intimacy or affiliation-intimacy or power or dominance or nurturance)

implicit and self-attributed affiliation motiv*

implicit and self-attributed intimacy motiv*

implicit and self-attributed motiv*

implicit and self-attributed power motiv*

implicit explicit congruenc*

implicit-explicit and (correlation or relation or relations or relationship or independence)

implicit explicit measur*

implicit explicit motiv*

implicit goals and motives

implicit intimacy

implicit motiv*

implicit motive and goal

implicit personality measur*

implicit power motivation and dominance

implicit power motivation and self reported dominance

implicit system

independence between needs and values and traits

intimacy motivation and congruenc*

intimacy motivation and (CPI or PRF)

intimacy motivation and explicit goa*

intimacy motivation and goa*

intimacy motivation and personality scal*

intimacy motivation and personal goa*

intimacy motivation and questionnair*

intimacy motivation and self-ratin*

intimacy motivation and self-repor*

intimacy motivation congruenc*

intimacy motivation explicit goa*

intimacy motivation goa*

intimacy motivation personality scal*

intimacy motivation personal goa*

intimacy motivation PRF

intimacy motivation questionnair*

intimacy motivation self-ratin*

intimacy motivation self-repor*

intimacy motive and congruenc*

intimacy motive and (CPI or PRF)

intimacy motive and explicit

intimacy motive and explicit goa*

intimacy motive and goa*

intimacy motive and personal goa*

intimacy motive and personality scal*

intimacy motive and questionnair*

intimacy motive and self-ratin*

intimacy motive and self-repor*

intimacy motive congruenc*

intimacy motive CPI

intimacy motive explicit

intimacy motive explicit goa*

intimacy motive goa*

intimacy motive personal goa*

intimacy motive personality scal*

intimacy motive PRF

intimacy motive questionnair*

intimacy motive self-ratin*

intimacy motive self-repor*

model of dual attitudes

most-memorable experiences

motiv* and (operant or needs) and (goal or goals or values or respondent or traits or sociotropy or autonomy or sociability or agency or communion) and (congruenc* or correlation or relation or relations)

motiv* and (projective or nonconscious) and (nonprojective or objective) and (measurement or assessment) and (congruenc* or correlation or relation or relations or relationship or independence)

motiv* and (projective or nonconscious) and (nonprojective or objective) and (congruenc* or correlation or relation or relations or relationship or independence)

motivational congruence

motivational congruences

motivational congruency

motivation and congruenc*

motivation implicit and explicit

motivation implicit explicit

motive and congruenc*

motives and congruences

motive and goal congruenc*

motive and goals congruence

motive dispositions and personal goals

motives and goal congruency

motives and goals congruency

motive congruenc*

motive-goal congruenc*

motive goal congruenc*

multitrait-multimethod validation of fear of success

narratives and self-knowledge

operant and respondent measures

operant and respondent personality measures

personal goals and social motives

personal motives and personality

power motivation and congruence

power motivation and congruences

power motivation and congruencies

power motivation and congruency

power motivation and CPI

power motivation and explicit

power motivation and explicit goa*

power motivation and goa*

power motivation and personal goa*

power motivation and personality scal*

power motivation and PRF

power motivation and questionnair*

power motivation and self-ratin*

power motivation and self-repor*

power motivation congruenc*

power motivation CPI

power motivation explicit

power motivation explicit goa*

power motivation goa*

power motivation personal goa*

power motivation personality scal*

power motivation PRF

power motivation questionnair*

power motivation self-ratin*

power motivation self-repor*

power motive and congruenc*

power motive and (CPI or PRF)

power motive and explicit

power motive and explicit goa*

power motive and goa*

power motive and personal goa*

power motive and questionnair*

power motive and self-ratin*

power motive and self-repor*

power motive congruenc*

power motive CPI

power motive explicit

power motive explicit goa*

power motive goa*

power motive personal goa*

power motive PRF

power motive questionnair*

power motive self-ratin*

power motive self-repor*

projective and questionnaire measures of achievement

projective and questionnaire measures of affiliation

projective and questionnaire measures of intimacy

projective and questionnaire measures of motiv*

projective and questionnaire measures of power

PSE and (CPI or PRF)

PSE and explicit

PSE and explicit goa*

PSE and explicit measur*

PSE and goa*

PSE and personal goa*

PSE and personality scal*

PSE and self-ratin*

PSE and self-repor*

PSE CPI

PSE explicit

PSE explicit goa*

PSE explicit measur*

PSE goa*

PSE personal goa*

PSE personality scal*

PSE PRF

PSE questionnair*

PSE self-ratin*

PSE self-repor*

questionnaire and implicit motive

questionnaire and implicit motives

questionnaire apperceptive

questionnaire implicit

questionnaire implicit motive

questionnaire motive

questionnaire PSE

questionnaire TAT

Relationships between achievement-related motives

self-attributed and implicit motiv*

strangers to ourselves

TAT and CPI

TAT and EPPS

TAT and explicit

TAT and explicit goal

TAT and explicit goals

TAT and objective personality assessment

(TAT or PSE) and goals

(TAT or PSE) and questionnaire

TAT and PRF

TAT and PSI

TAT CPI

TAT explicit

TAT goal

TAT goals

TAT personal goal

TAT personal goals

TAT personality scale

TAT personality scales

TAT PRF

TAT questionnaire

TAT self-rating

TAT self-ratings

TAT self-report

TAT self-reports

thematic apperception and (CPI or PRF)

thematic apperception and personal goa*

thematic apperception and questionnair*

thematic apperception and self-ratin*

thematic apperception and self-repor*

thematic apperception CPI

(thematic apperception or thematic analysis or picture story or TAT or picture-story or picture-story exercise or Picture Story Exercise or PSE) and (explicit measur* or questionnaire or personality scal* or inventory or self-ratin* or self-repor* or self-evaluation or self evaluation or personal strivin* or goal or goals or Personality Research Form or PRF or California Psychological Inventory or CPI or EPPS or Edwards Personal Preference Schedule or personal striving assessment packet or PSAP)

thematic apperception personal goa*

thematic apperception personality scal*

thematic apperception PRF

thematic apperception questionnair*

thematic apperception self-ratin*

thematic apperception self-repor*

thematic apperception test and questionnaire measures

two approaches to motivation

unwanted goal

unwanted goals
